# DNA methylation reprogramming in cancer: Does it act by re-configuring the binding landscape of Polycomb repressive complexes?

**DOI:** 10.1002/bies.201300130

**Published:** 2013-11-26

**Authors:** James P Reddington, Duncan Sproul, Richard R Meehan

**Affiliations:** MRC Human Genetics Unit, MRC IGMM, University of EdinburghEdinburgh, United Kingdom

**Keywords:** cancer epigenetics, DNA methylation, epigenomics, H3K27me3, Polycomb, reprogramming

## Abstract

DNA methylation is a repressive epigenetic mark vital for normal development. Recent studies have uncovered an unexpected role for the DNA methylome in ensuring the correct targeting of the Polycomb repressive complexes throughout the genome. Here, we discuss the implications of these findings for cancer, where DNA methylation patterns are widely reprogrammed. We speculate that cancer-associated reprogramming of the DNA methylome leads to an altered Polycomb binding landscape, influencing gene expression by multiple modes. As the Polycomb system is responsible for the regulation of genes with key roles in cell fate decisions and cell cycle regulation, DNA methylation induced Polycomb mis-targeting could directly drive carcinogenesis and disease progression.

## Introduction

Epigenetic systems modulate the interpretation of the information contained in genomes by regulating DNA dependent processes. Many different epigenetic pathways act in mammalian cells and recent work has highlighted extensive interactions between them 1. The epigenome is frequently altered in cancer, and there is evidence to suggest that interactions between distinct epigenetic mechanisms are important drivers in this process 2,3. Identifying the causes of epigenetic reprogramming in cancer and understanding its consequences for disease pathology are key challenges in molecular biology.

DNA methylation and the Polycomb repression system are two epigenetic pathways that play key roles in cancer formation and progression. Here, we focus on recent evidence that links DNA methylation to the genomic targeting of Polycomb repressive complexes (PRCs), the effectors of the Polycomb system. In light of this evidence, we discuss how widespread reprogramming of DNA methylation patterns in cancer could drive the relocation of PRCs on chromatin, and contribute to carcinogenesis through alterations in gene expression.

## The cancer DNA methylome: How does it change and what are the consequences?

**Figure 1:**
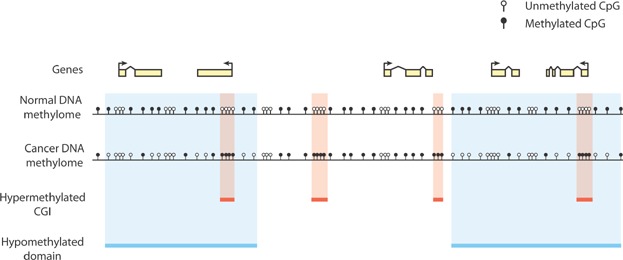
Reprogramming of DNA methylation patterns in cancer. Frequently observed changes to the DNA methylome in cancer are depicted for a portion of a hypothetical genome. CpG dinucleotides are depicted as open (unmethylated cytosine) or filled (5-methylcytosine). In healthy somatic cells (“Normal DNA methylome”) the background level of CpG methylation throughout the bulk genome is high, while CGIs are infrequently methylated 5,28. In cancer, CGIs frequently become hypermethylated (red bars and boxes), and CpG methylation is reduced across large genomic domains (blue bars and boxes) 14,16. Focal CGI hypermethylation frequently occurs within larger hypomethylated domains 16. Only unmethylated and 5-methylcytosine are shown for simplicity. CGI, CpG island.

Mammalian genomes exhibit widespread DNA methylation that is acquired during early development 4. DNA methylation is laid down and maintained by the DNA methyltransferases, DNMT1, DNMT3A, and DNMT3B, and generally occurs at cytosines in a 5′-CG-3′ (CpG) dinucleotide context 4. This pervasively methylated landscape is interspersed with short hypomethylated regions, the best described of which are CpG islands (CGIs) 5 ( Fig. 1). Enzymes of the TET family can oxidize methylated cytosine (mC) to generate further modified bases, of which 5′-hydroxymethylcytosine (hmC) is the most abundant 6. The oxidation of mC by TET enzymes is proposed to be the first step in DNA demethylation pathways but stable levels of hmC of unknown significance are found in the bodies of active genes 6.

DNA methylation is classically implicated in gene regulation at promoters, where it is proposed to repress transcription initiation by attenuating the binding of transcription factors, and recruiting repressor complexes through the attraction of methyl-CpG binding proteins 7. CGIs rarely become methylated during normal development and most are likely to function as gene promoters 5. However, the methylation of some CGIs plays a vital role in genomic imprinting, the inactivation of one copy of the X chromosome in female cells and the regulation of some tissue-specific genes 4.

## Cancer DNA methylomes exhibit focal hypermethylation and widespread hypomethylation

Two radical differences exist between normal mammalian DNA methylation landscapes and those found in cancer: many CGIs become aberrantly hypermethylated in cancerous cells, and large amounts of DNA methylation are lost from other genomic regions 8 ( Fig. 1). Interestingly, the Polycomb system has been suggested to play an instructive role in the hypermethylation of promoter DNA in cancer, as genes that are subject to promoter hypermethylation are frequently marked by PRC2-deposited H3K27me3 in early development 2,9,10. Hypermethylation of CGI promoters was classically believed to directly drive carcinogenesis by silencing tumor suppressor genes 8. Studies using genomic profiling technologies have conclusively demonstrated that this is not the case for the vast majority of aberrantly hypermethylated genes, as they are already silenced in the corresponding normal cells that give rise to cancer 11–13. However, these observations do not rule out the possibility that rare driver genes are directly silenced by aberrant CGI hypermethylation 11.

In addition to aberrant CGI hypermethylation, cancer genomes exhibit widespread loss of DNA methylation ( Fig. 1). Work over many years suggested that loss of methylation occurs primarily in the repetitive portion of the genome, in particular satellite repeats and LINE-1 retrotransposons 14. This view has been radically altered by the application of whole genome bisulfite sequencing technology to cancer, which revealed that DNA methylation is lost from megabase-scale genomic domains 3,15,16. Although these domains are enriched in LINE-1 elements, those lying outside of these domains do not become hypomethylated, suggesting that the loss of methylation is not specific to retrotransposons 14,15. Hypomethylated domains often overlap with other features of mammalian genomes, particularly lamin-associated domains, which associate with the nuclear lamina at the periphery of the nucleus 14,16. However, it is currently unclear whether the nuclear lamina is mechanistically connected to the loss of methylation in cancer.

Experiments in transgenic animals have demonstrated that genomic hypomethylation can either inhibit or promote carcinogenesis depending on the cellular context 17,18. Loss of DNA methylation causes genomic instability in transgenic animals 17, cultured cells 19, and in patients with Immunodeficiency–Centromeric instability–Facial anomalies (ICF) syndrome, which is caused by mutations in *DNMT3B* 20. Hypomethylation may also promote cancer by potentiating the activation and transposition of LINE-1 elements 21. Such activation can potentially directly disrupt genes through transposition into their vicinity 22,23 or because transcription from LINE-1 promoters can affect neighboring genes 24,25.

Despite the application of the latest genomic approaches to cancer DNA methylomes, we still have a limited understanding of the causes of DNA methylation reprogramming and its impact on cancer phenotypes. The picture is complicated by heterogeneity caused by differences in cellular origin or stochastic clonal evolution 12. Furthermore, recent studies seeking to understand the role of DNA methylation in normal cells have uncovered previously unappreciated functions of the DNA methylome in gene regulation, highlighting the diverse ways that this epigenetic mark is utilized in mammalian genomes 1.

## A new role for DNA methylation in shaping the Polycomb landscape

In addition to methyl-CpG binding proteins, we now know that an eclectic variety of proteins exhibit DNA methylation-modulated binding to chromatin 1,26. Many of these possess histone-modification activity, and are involved in setting up diverse aspects of chromatin organization, highlighting roles for DNA methylation outside of promoter proximal regions 1. This raises the possibility that the effects of an altered DNA methylome in cancer could be more widespread than currently appreciated.

## DNA methylation attenuates PRC2 binding to chromatin

It has emerged that DNA methylation plays an unexpected role in restricting the genomic targeting of Polycomb repressor complexes (PRCs) (see Box 1 for an overview of Polycomb repressive complexes and their targeting). While DNA methylation and Polycomb were once considered to be two independent and complementary pathways of transcriptional repression, it is now conceivable that direct cross talk occurs between them. Epigenome profiling experiments showed that high levels of DNA methylation and H3K27me3 are rarely found at the same loci in mammalian genomes, suggesting that the presence of one mark is antagonistic to the other 27–32. This relationship is most prominent at CGIs 31, but can be observed to a lesser extent at large partially methylated domains (PMDs) in cultured cells 28,30. Exclusivity between the two marks is exemplified by the imprinted *Rasgrf1* locus where one allele is marked by DNA methylation and the other by H3K27me3 33.

**Box 1 – Polycomb repressive complexes and their genomic targeting**

The Polycomb system is a highly conserved epigenetic mechanism that contributes to the stable repression of thousands of target genes outside of their normal expression domains 40. The system comprises the coordinated action of several multi-protein complexes that associate with chromatin, many of which chemically modify histone proteins through the deposition of histone marks. The Polycomb Repressive Complex 2 (PRC2) catalyses tri-methylation of lysine 27 on histone H3 (H3K27me3), a signature of repression mediated by this complex. A subset of Polycomb repressive complex 1 (PRC1) binds to the H3K27me3 mark and catalyses the mono-ubiquitination of histone H2A. A major question in Polycomb research is how PRCs are targeted to the correct genomic compartments 40. Polycomb targeting is best understood in the fruit fly, where PRCs are recruited to specific sequence elements called Polycomb response elements (PREs) by combinations of sequence-specific binding proteins 40. PRC recruitment is far less understood in mammals and is thought to result from the interaction between multiple DNA sequence features and chromatin structure 40. For example, CGIs have been linked to the recruitment of Polycomb complexes 36, but the mechanism of recruitment to these elements remains unclear.

Recent studies have addressed the cause-consequence relationships involved in establishing these patterns by perturbing either DNA methylation or H3K27me3 and asking what happens to the other mark. In multiple organisms and experimental systems, the removal of DNA methylation has a profound influence on the distribution of the H3K27me3 mark throughout the genome 29,31,33–37. Crucially, removal of DNA methylation results in accumulation of the PRC2 complex and H3K27me3 in illegitimate genomic locations that were previously DNA methylated 31,34,35, suggesting that dense DNA methylation is capable of attenuating PRC2 binding to chromatin. This is supported by in vitro experiments demonstrating reduced PRC2 occupancy and activity on DNA methylated chromatin templates 26,34. In addition, TET1 is required for a significant proportion of PRC targeting in mouse ES cells, connecting this putative demethylation pathway to PRC recruitment 38. In contrast, when PRC2 components are removed only modest changes in DNA methylation are observed 39, suggesting that the H3K27me3 mark does not have a similar reciprocal effect on the placement of DNA methylation in non-transformed cells.

While the majority of studies have focused on the influence of DNA methylation on the PRC2 complex, it is likely that PRC1 localization is also affected. Canonically, PRC1 is recruited to genomic loci by the H3K27me3 mark laid down by PRC2 40, so restriction of PRC2 binding by DNA methylation would be expected to also affect PRC1 recruitment. A recent study has also detailed a non-canonical PRC1 recruitment pathway mediated by the KDM2B protein, which contains an unmethylated CpG binding CXXC domain 41.

## The DNA methylome is required for correct PRC2-mediated gene repression

As PRCs are involved in transcriptional repression, their redistribution upon loss of DNA methylation can have significant effects on the transcriptome. For example, in *Dnmt3a* mutant neural stem cells, levels of DNA methylation are reduced within the body of some actively transcribed genes, leading to PRC2 binding and repression of their transcription 34. Removal of most DNA methylation from mouse embryonic fibroblasts (MEFs) leads to a variety of transcriptional consequences connected to PRC redistribution 35. Genes lying within regions of the genome that accumulate H3K27me3 in DNA methylation mutants are often transcriptionally down-regulated, consistent with de novo repression by PRC2 within these regions 35. Surprisingly, many normal PRC2 target genes are de-repressed in DNA methylation mutants, concomitant with loss of H3K27me3 from their promoter regions 35. Importantly, these genes are associated with unmethylated CGI promoters in wild type cells 35, meaning that DNA methylation would not normally be implicated in their regulation. The loss of H3K27me3 observed here could be explained by dilution of a limited amount of PRC2, due to the increased binding of this complex to numerous intergenic sites uncovered by loss of DNA methylation 35.

Many interesting questions remain concerning the relationship between DNA methylation and the Polycomb system and its implications for genome regulation. Despite the fact that in vitro experiments have suggested that PRC2 is able to directly read CpG methylation states 26,34, the molecular mechanism underlying this cross talk is currently furtive. One important implication of these observations is that reprogramming of DNA methylation patterns in cancer could trigger mis-regulation of transcriptional programs through subsequent redistribution of the repressive activity of PRCs.

## Do DNA methylation changes drive Polycomb redistribution in cancer?

In addition to changes in the DNA methylome, H3K27me3 patterns are subject to reprogramming in cancer cells 32,42. Studies have documented correlated changes in DNA methylation and H3K27me3 in cancer, raising the possibility that at least some of the redistribution of these two marks is causally linked 3,43.

**Figure 2:**
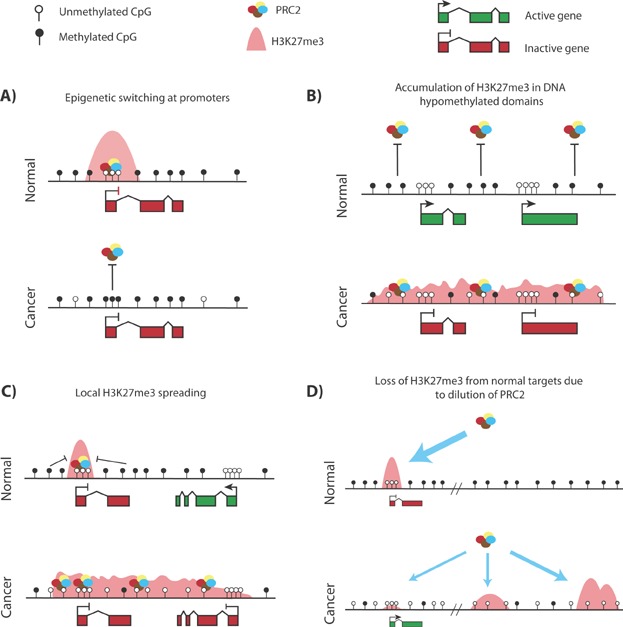
Putative pathways for gene mis-regulation in cancer. Previously described and hypothetical changes to PRC2-mediated gene repression in cancer in response to DNA methylation redistribution. A: DNA hypermethylation of a PRC2-bound promoter CGI in cancer causes loss of PRC2 and H3K27me3 43. B: The loss of DNA methylation across large genomic domains could allow PRC2 to form large blocks of H3K27me3 modified chromatin de novo, leading to the silencing of multiple adjacent genes 3. C: Loss of DNA methylation surrounding H3K27me3 marked regions allows spreading of PRC2 occupancy into adjacent chromatin and de novo gene repression. D: Widespread loss of DNA methylation throughout the cancer genome allows PRC2 to occupy a large number of new sites, distributing the complex over a larger proportion of the genome. As a result, PRC2 occupancy is reduced at normal PRC2-target genes, leading to de-repression of a subset of them that are particularly sensitive to PRC2 reduction 35. PRC2, Polycomb repressive complex 2.

Based on frequently observed changes to the DNA methylome in cancer, multiple putative effects on PRC-mediated gene regulation can be envisaged ( Fig. 2). One such pathway is epigenetic switching, where DNA methylation replaces PRCs at CGIs in cancer 43 ( Fig. 2A). Both studies of individual genes and epigenomic profiling have shown that promoter CGIs that are subject to aberrant hypermethylation in cancer are frequently marked by PRCs during development 2,9,10. In prostate cancer, developmentally important CGI genes silenced by PRC2 in normal prostate cells acquire DNA methylation concomitant with loss of their PRC2 marks 43. Because these genes are normally silent in the tissue that gives rise to cancer, this epigenetic switch would not be expected to cause de novo repression. However, as repression by DNA methylation is more stable than PRC-mediated repression, this switch could significantly reduce epigenetic plasticity by preventing the future activation of genes in response to external stimuli, potentially blocking cellular differentiation 11.

Genomic hypomethylation in cancer is also likely to impact on the transcriptome. The de novo formation or exacerbation of PMDs in cancer, due to the processes underlying hypomethylation, could uncover binding sites for PRC2, allowing the formation of new H3K27me3 domains and causing the repression of multiple adjacent genes ( Fig. 2B) 3. The H3K27me3 modification is thought to be propagated by the binding of the PRC2 component EED to H3K27me3 resulting in spreading of PRCs unless boundaries are applied 44. DNA methylation surrounding a PRC2-bound locus could act locally to prevent spreading of H3K27me3 into neighboring regions ( Fig. 2C). Proximity to existing H3K27me3 marked loci could, therefore, play an important role in determining whether a gene becomes repressed by PRC recruitment following loss of DNA methylation.

In addition to de novo gene repression, loss of PRC restriction by DNA methylation in cancer could paradoxically result in the de-repression of Polycomb-target genes, as observed in DNA methylation mutant MEFs 35 ( Fig. 2D). In support of this hypothesis, loss of H3K27me3 and de-repression of PRC targets has been reported in cancer 45,46, but it is currently unclear if these alterations are driven by cancer-associated hypomethylation. Such a pathway is most probable in cancers that exhibit the largest degree of DNA hypomethylation. However, the picture is likely to be complicated by the elevated expression, and frequent mutation, of Polycomb components in certain cancers 40,42. It has also been suggested that the relationship between DNA methylation and PRC2 is fundamentally different in normal and transformed cells, adding a further layer of complexity to this problem 29. The targets of PRCs include a large number of genes with key functions in cell lineage decisions and the regulation of the cell cycle 40. The mis-regulation of these genes could in theory have a major impact on the formation and progression of cancer, providing the impetus for further research in this area.

## Conclusions and prospects

In differentiated cells part of the barrier to transformation is precise partitioning of the genome into active and repressed domains; epigenetic reorganization of these domains is a feature of both cellular and cancer reprogramming. Understanding the cause and functional impact of epigenetic reprogramming is a major goal of both basic and clinical research. The action of PRCs is fundamentally important to many types of cancers as exemplified by the recurrent mutations of PRC components uncovered by cancer re-sequencing studies 14. Reprogramming of the DNA methylome in cancer could drive further epigenetic instability in an unexpected way, by reshaping the PRC binding landscape.

The functional consequences of DNA methylation mediated PRC redistribution as described here are also likely to be important in other biological systems. Large-scale hypomethylation and the formation of PMDs are now known to be a feature of aging cells 47 and of some normal cell populations including the placenta 48 and mature B-cells 49.

In cancer, this mechanism could potentially impact genome regulation in many ways, leading to a transcriptome that facilitates cancer formation, plasticity, and progression, or influences how cancers respond to therapy. We are just beginning to comprehend the epigenetic heterogeneity that exists in cancer 14. Changes to the DNA methylome driven by a stochastic or step-wise process could offer a substrate for cellular Darwinism 14,50, providing intermediates with favorable patterns of gene expression that arise due to altered H3K27me3 targeting. Future studies should concentrate on dissecting the cause-consequence relationships involved, and exploring potential points of therapeutic intervention.

## Abbreviations

CGI: CpG island
CpG: cytosine followed immediately by a guanine in the 5′ to 3′ direction
ES: embryonic stem (cells)
H3K27me3: tri-methylation of lysine 27 on histone H3
hmC: 5′-hydroxymethylcytosine
LINE: long interspersed nuclear element
mC: 5′-methylcytosine
MEFs: mouse embryonic fibroblasts
PMDs: partially (DNA) methylated domains
PRC: Polycomb repressive complex
PRC1: Polycomb repressive complex 1
PRC2: Polycomb repressive complex 2
